# Targeted Muscle Reinnervation and Regenerative Peripheral Nerve Interfaces Versus Standard Management in the Treatment of Limb Amputation: A Systematic Review and Meta-Analysis

**DOI:** 10.1177/22925503221107462

**Published:** 2022-06-16

**Authors:** Morgan Yuan, Matteo Gallo, Lucas Gallo, Minh HQ Huynh, Mark McRae, Matthew C. McRae, Achilleas Thoma, Christopher J. Coroneos, Sophocles H. Voineskos

**Affiliations:** 1 Michael G. DeGroote School of Medicine, 3710 McMaster University, Hamilton, Canada; 2 Faculty of Medicine, 12365 University of Ottawa, Ottawa, Canada; 3 Division of Plastic Surgery, Department of Surgery, 3710 McMaster University, Hamilton, Canada; 4 Department of Health Research Methods, Evidence and Impact (HEI)

**Keywords:** plastic surgery, systematic review, amputation, neuroma, peripheral nerve, targeted muscle reinnervation, Chirurgie plastique, étude systématique, amputation, névrome, nerf périphérique, réinnervation musculaire ciblée

## Abstract

**Introduction:** Painful neuromas are a common postoperative complication of limb amputation often treated with secondary reinnervation. Surgical reinnervation include Targeted Muscle Reinnervation (TMR) and Regenerative Peripheral Nerve Interface (RPNI), and can be primary and secondary. The aim of this review is to assess the effects of primary TMR/RPNI at the time of limb amputation on the incidence and intensity of post-operative neuroma and pain. **Methods:** This review was registered *a priori* on PROSPERO (CRD42021264360). A search of the following databases was performed in June 2021: Medline, EMBASE, and CENTRAL. Unpublished trials were searched using clinicaltrials.gov. All randomized and non-randomized studies assessing amputation with a reinnervation strategy (TMR, RPNI) were included. Outcomes evaluated included the incidences of painful neuroma, phantom limb pain (PLP), residual limb pain (RLP), as well as severity of pain, and Pain intensity, behavior, and interference (PROMIS). **Results:** Eleven studies were included in this systematic review, and five observational studies for quantitative synthesis. Observational study evidence suggests that TMR/RPNI results in a statistically significant reduction in incidence, pain scores and PROMIS scores of PLP and RLP. Decreased incidence of neuromas favored primary TMR/RPNI, but this did not achieve statistical significance (p = 0.07). Included studies had moderate to critical risk of bias. **Conclusion:** The observational data suggests that primary TMR/RPNI reduces incidence, pain scores and PROMIS scores of PLP and RLP. Going forward, randomized trials are warranted to evaluate this research question, particularly to improve the certainty of evidence.

## Résumé

**Introduction:** Les névromes douloureux sont une complication postopératoire courante de l’amputation d’un membre qui est souvent traitée par réinnervation secondaire. La réinnervation chirurgicale inclut la réinnervation musculaire ciblée (TMR) et l’interface nerveuse périphérique régénérative (RPNI) qui peuvent être primaires ou secondaires. Le but de cette revue était d’évaluer les effets de la TMR/RPNI primaires au moment de l’amputation du membre sur l’incidence et l’intensité du névrome et de la douleur postopératoire. **Méthodes:** Cette revue a été enregistrée *a priori* sur PROSPERO (CRD42021264360). Une recherche a été réalisée en juin 2021 dans les bases de données suivantes : MEDLINE, EMBASE et CENTRAL. Les essais non publiés ont été recherchés à l’aide du site clinicaltrials.gov. Toutes les études randomisées et non randomisées évaluant l’amputation avec stratégie de réinnervation (TMR, RPNI) ont été incluses. L’évaluation des résultats a inclus l’incidence des névromes douloureux, des douleurs du membre fantôme (PLP), de douleur résiduelle du membre (RLP) ainsi que la sévérité de la douleur, l’intensité de la douleur, le comportement et l’interférence (PROMIS). **Résultats:** Onze études ont été incluses dans cette revue systématique et cinq études observationnelles pour la synthèse quantitative. Les données probantes d’étude observationnelle suggèrent que la TMR/RPNI entraîne une réduction statistiquement significative de l’incidence, des scores de douleur et des scores PROMIS des PLP et RLP. La baisse de l’incidence des névromes favorisait les TMR/RPNI primaires, mais sans atteindre la signification statistique (*P *= 0,07). Les études incluses comportaient des risques de biais modérés à critiques. **Conclusion:** Les données observationnelles suggèrent que les TMR/RPNI réduisent l’incidence, les scores de douleur et les scores PROMIS de PLP et RLP. La réalisation d’essais randomisés est justifiée pour aller de l’avant et évaluer cette question de recherche, en particulier pour améliorer la certitude des données probantes.

## Introduction

Limb amputations are one of the oldest surgical procedures and have been used as a life-saving treatment for thousands of years.^1^ Despite improvements in surgical techniques, experience, and instrumentation, there continues to be significant morbidity and pain associated with major extremity amputation.^2^ Approximately half all amputees continue to experience some form of residual or phantom limb pain in the months following their operation and approximately one third develop painful neuromas.^3–5^ These complications will often necessitate additional surgeries and secondary reinnervation strategies in an attempt to prevent chronic pain.

Prior to an amputation, nerves are completely encased in an epineural sheath composed of well-organized fibrous connective tissue. When this sheath is significantly disrupted, such as during amputation, the previously enclosed axons are able to proliferate into surrounding scar and connective tissue and lead to the development of symptomatic neuromas.^6^ Reinnervation strategies attempt to treat neuromas by implanting the resected nerve into nearby healthy muscle tissue and create a better environment for more organized reinnervation. The two most common techniques for doing so are Targeted Muscle Reinnervation (TMR) and Regenerative Peripheral Nerve Interface (RPNI).^7^ TMR is a procedure which is increasingly being used to treat symptomatic neuromas by using a nearby healthy muscle segment as a conduit for more organized axonal proliferation.^8^ Similarly, RPNI involves taking a free muscle graft from the amputated limb (either proximal or distal to the site of amputation) and wrapping the grafted muscle around the dissected nerve allowing for more controlled nerve growth to motor end plates on the grafted tissue.^9^ In both of these procedures, the nerve is able to reinnervate and remodel within a denervated muscle segment. This provides many targets for reinnervation when compared to nearby intact muscles and reduces the likelihood of disorganized axonal sprouting which can occur when a nerve is left in fibrotic scar tissue. This subsequently decreases the risk of neuroma formation and the potential for pain following amputation.^6,8–10^

Although these surgeries are typically performed in response to the development of painful complications following amputation, these same reinnervation strategies can be employed prophylactically at the time of the amputation to prevent the formation of painful neuromas.^7^ To our knowledge, there have been no previous systematic reviews comparing the efficacy of primary TMR and RPNI at the time of amputation with the standard of care at decreasing complications and preventing the need for revisional surgery. Therefore, the aim of this review is to assess the effects of primary TMR and RPNI at the time of limb amputation on the incidence and intensity of post-operative neuroma and pain.

## Methods

### Types of Studies

This review included all-language, randomized, quasi-randomized and comparative observational studies.

### Types of Participants

Studies involving male or female participants greater than 18 years of age, in any clinical setting, who underwent primary TMR and/or RPNI at the time of upper or lower limb amputation were included in this study.

### Types of Intervention

Any primary reinnervation strategy (ie TMR or RPNI) performed at the time of upper or lower limb amputation was analyzed. Reinnervation must have been applied “prophylactically”, in the intraoperative or immediate postoperative setting. Reinnervations performed as a result of complications were excluded. The comparator group included any amputation that did not use a reinnervation strategy during or after amputation which is considered to be the current standard of treatment.

### Types of Outcomes

A list of outcomes was generated based on existing systematic reviews and primary observational studies identified through a preliminary search of the literature. Five outcomes deemed to be critical and/or important were included in this review. Outcomes selected include: 1) Incidence of neuroma; 2) Incidence of phantom limb pain (PLP); 3) Incidence of residual limb pain (RLP); 4) Severity of Pain; 5) Pain intensity, behavior, and interference for PLP and RLP (ie, PROMIS).

The study authors’ definition of these outcomes was accepted. For this review, any duration of follow-up reported as the study's primary time-horizon was accepted; if not specified, data from the final time horizon was collected.

### Search Methods for Identification of Studies

This trial was registered a priori on PROSPERO (CRD42021264360). A search of the following databases was performed on June 1, 2021 with the assistance of a health sciences librarian without language restriction:

- OVID Medline Epub Ahead of Print, In-Process & Other Non-indexed Citations, OVID MEDLINE(R) Daily and Ovid MEDLINE (R) 1946 to June 2021.
- EMBASE 1974 to June 2021.
- The Cochrane Central Register of Controlled Trials, CENTRAL (up to June 2021).

Details of the specific search strategies can be found within the supplementary appendix 1. Ongoing trials were identified using the WHO international clinical Trials Registry Platform and ClinicalTrials.gov database (up to June 2021). Trialists of ongoing studies were contacted, as needed, for further data. A hand search of the reference list of included studies was performed to screen for relevant articles.

### Data Extraction

Two independent review authors screened, extracted data, and assessed the risk of bias of included studies using piloted forms. All disagreements were settled by discussion between the review authors. Authors of studies were contacted if insufficient information was available in the manuscript.

### Risk of Bias Assessment

Risk of bias of included randomized and non-randomized studies was performed using recommendations in accordance with the Cochrane Handbook for Systematic Reviews of Interventions Version 6.1.^11^ Studies deemed to be individually randomized were assessed using the Cochrane Risk of Bias tool (RoB 2.0).^12^ Risk of bias within eligible non-randomized studies was evaluated using the Risk of Bias in Non-randomized Studies – of Interventions (ROBINS-I) assessment tool.^13,14^ Risk of bias was summarized using a “Risk of Bias’ table.

The Grading of Recommendations Assessment, Development and Evaluation (GRADE) approach was used to evaluate the certainty of the evidence for each outcome. Certainty was classified as “high”, “moderate”, “low”, or “very low” according to the GRADE domains.^11^ GRADEpro software was used to summarize the certainty of the evidence and magnitude of effect for each outcome using a “Summary of Findings’ table.^15^

### Data Synthesis

The inverse variance method and random-effects model was used for the presentation of results in the form of a meta-analysis. Dichotomous outcomes were synthesized and reported as an odds ratio (OR) with 95% confidence intervals (CI). When ORs were not provided, the raw data was used to calculate the OR and 95% CI using Review Manager 5 [Review Manager (RevMan) Version 5.3].^16^ Continuous outcomes were summarized using standardized mean differences (ie, if different outcome measures were used) or mean differences (ie, if a single outcome measure was used) with 95% confidence intervals. All included studies utilized individual patients as the unit of analysis. For studies where ≥ 1 event occurred in one group and 0 events occurred in the other group, a value of 0.5 was imputed in the cell with 0 events to allow for the calculation of an OR.^11^

Heterogeneity of pooled results were evaluated using a combination of visual inspection (ie, magnitude of point estimates and overlapping CI of the forest plot) and statistical analyses (Chi^2^ test with significance set at p ≤ 0.10 and I^2^ statistic).^17^ The magnitude of heterogeneity as defined by the I^2^ value was: 0-40% as “might not be important”, 30-60% as “moderate” heterogeneity, 50-90% as “substantial” heterogeneity, and 75-100% representing “considerable” heterogeneity.^18^

Given that <10 studies were included for each outcome, a funnel plot was not used to evaluate the presence of publication bias.^19^ The WHO Trial Registry Platform and ClinicalTrials.gov databases were searched to evaluate the protocols of included studies for selective outcome reporting.

### Subgroup Analysis

A priori sensitivity analyses were planned to evaluate the impact on the pooled results and measures of heterogeneity by removing studies deemed to be at overall “high risk of bias’ from the analysis; however, this was not performed due to a limited number of included studies.

## Results

**Figure 1.**
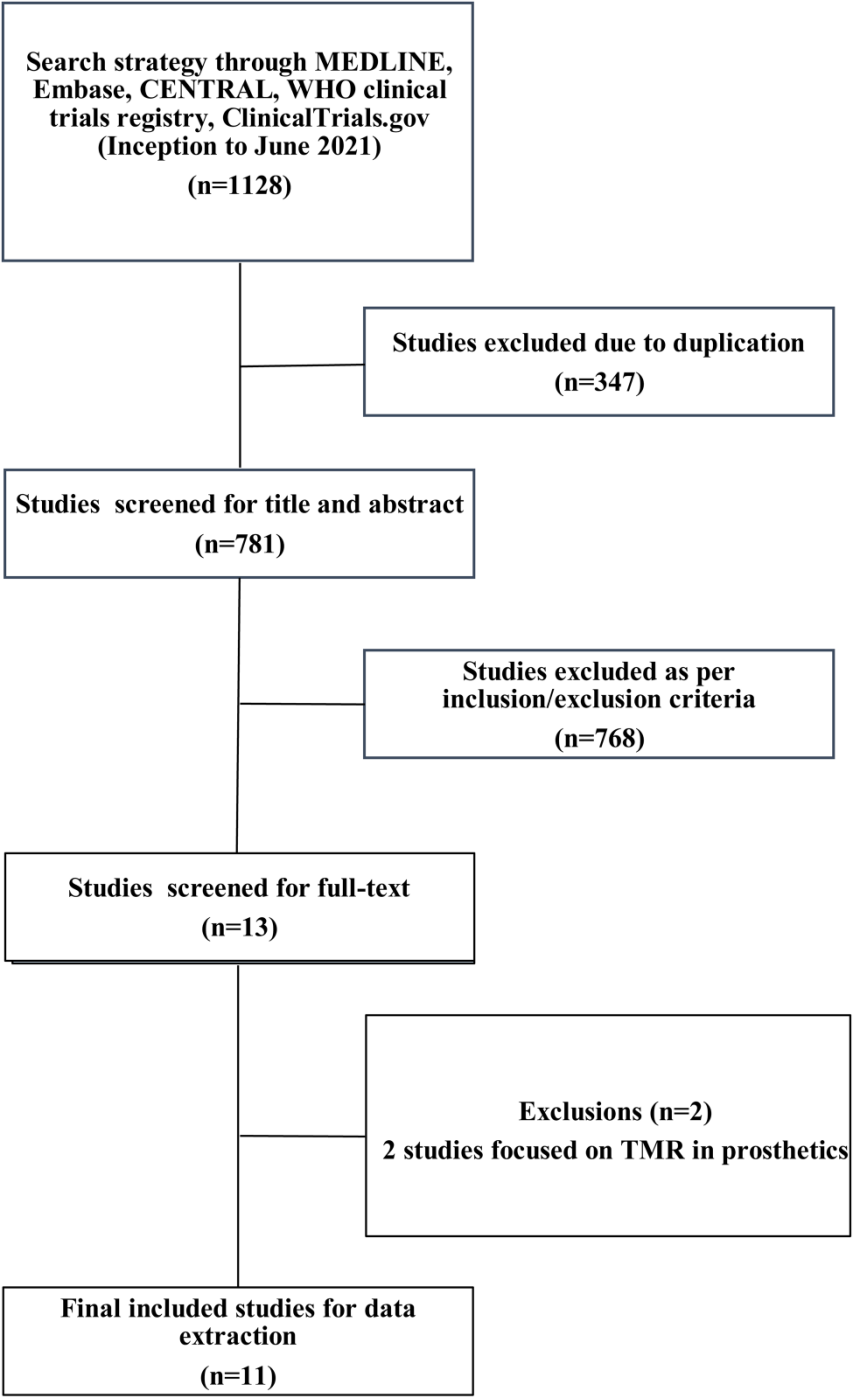
PRISMA Diagram of Included Studies.

The search strategy identified 1128 studies. After 347 studies were removed as duplicates, 781 studies were screened at title and abstract. Thirteen studies were then reviewed at full-text screening, with two studies that did not meet inclusion criteria. Eleven studies were included in the present systematic review (Table 1),^20–30^ with five studies^20–24^ summarized through meta-analysis. The details of the screening process are found in the PRISMA flow diagram (Figure 1).

**Table 1.** Characteristics of Included Studies.

Author	Year	Study Type	Intervention	Control	Sample size	Outcomes Assessed
Pet et al	2014	Retro Cohort	Primary TMR	Secondary TMR	35 (12 vs 23)	Incidence of neuroma, Incidence of phantom limb pain
Janes et al	2020	Retro Cohort	Primary TMR	Secondary TMR	17 (3 vs 10; 1 patient non amputee, 3 LTFU)	Incidence of neuroma
Hoyt et al	2021	Retro Cohort	Primary TMR or RPNI	Secondary TMR or RPNI	87 (28 vs 59)	Pain score
Valerio et al	2019	Pro Cohort	Primary TMR	Standard of Care	489 (51 vs 438)	Incidence of phantom limb pain, Incidence of residual limb pain, Pain score, PROMIS
Kubiak et al	2019	Retro Cohort	Primary RPNI	Standard of Care	90 (45 vs 45)	Incidence of neuroma, Incidence of phantom limb pain
Stoehr et al	2020	Case Series	Primary TMR	None	4 (no control)	Incidence of phantom limb pain, Pain score, PROMIS
Frantz et al	2020	Case Series	Primary TMR	None	25 (25 vs 0;no control)	Pain score, PROMIS
O’Brien et al	2021	Retro Cohort	Primary TMR	Standard of Care	71 (16 vs 55)	Incidence of phantom limb pain, Incidence of residual limb pain, Pain score, PROMIS
Anderson et al	2020	Case Series	Primary TMR	None	12 (11vs 0;1 LTFU, no control)	Incidence of neuroma, Incidence of phantom limb pain
Alexander et al	2019	Retro Cohort	Primary TMR	Standard of Care	89 (31 vs 58)	PROMIS
Chang et al	2021	Retro Cohort	Primary TMR	Standard of Care	200 (100/100)	Incidence of neuroma, Incidence of phantom limb pain, Incidence of residual limb pain

All five of the studies included in this review for quantitative synthesis were non-randomized studies, and represented 1061 patients. This included 283 patients who underwent primary TMR/ RPNI, and 778 patients that had either secondary TMR/RPNI (n = 82) or normal amputations (n = 696). Two observational studies were not included in this review for quantitative synthesis; one study^25^ evaluated PLP among secondary TMR/RPNI patients as the comparator group and one study^26^ did not provide PROMIS scores by intervention group. Additionally, three studies did not have an appropriate comparison group and therefore, were not pooled using a meta-analysis.

### Neuromas

**Figure 2.**
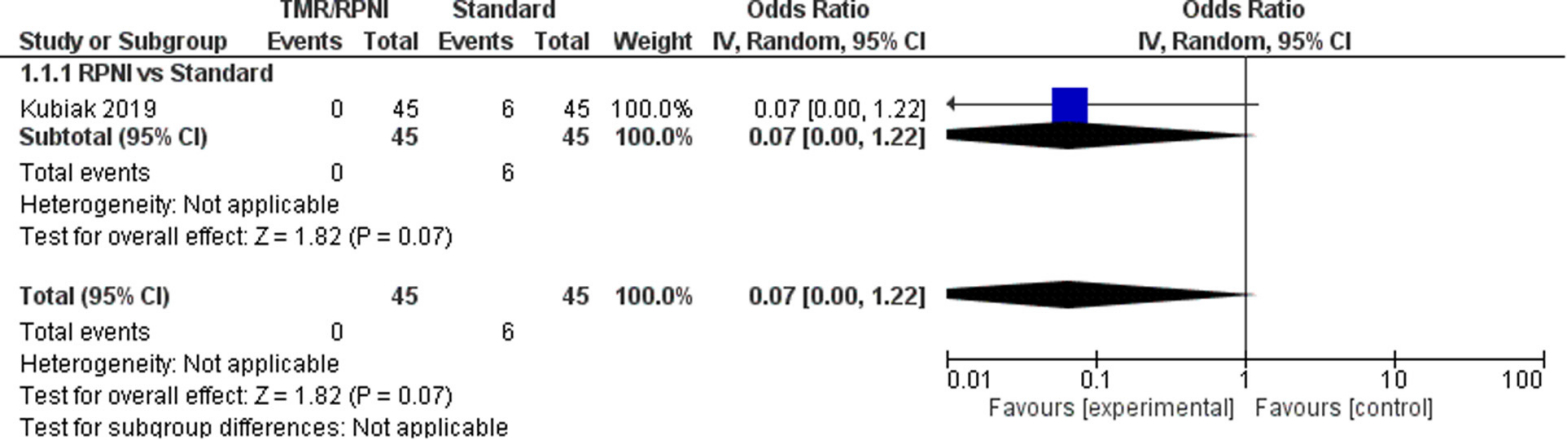
Forest Plot of Neuromas.

Only one observational study^22^ was identified, representing 90 patients, to be included for quantitative analysis for incidence of neuroma (Figure 2). From this, the odds ratio (OR) was calculated to be 0.07 (95% CI 0.00 to 1.22). There was moderate risk of bias in the identified study due to potential bias in confounding and measurement of outcome. The overall certainty of evidence was low. In summary, the study evidence suggests that TMR/RPNI may result in a large reduction in incidence of neuroma formation, but is limited in this study due to evidence from only one study.

In addition, two observational studies^24,25^ and two case series^27,30^ evaluated the incidence of neuromas in TMR/RPNI patients only. In total, these five studies represented 171 patients, with a pooled neuroma incidence in TMR/RPNI patients of 2.34% (individual study incidence range: 0% to 9.09%).

### Phantom Limb Pain

**Figure 3.**
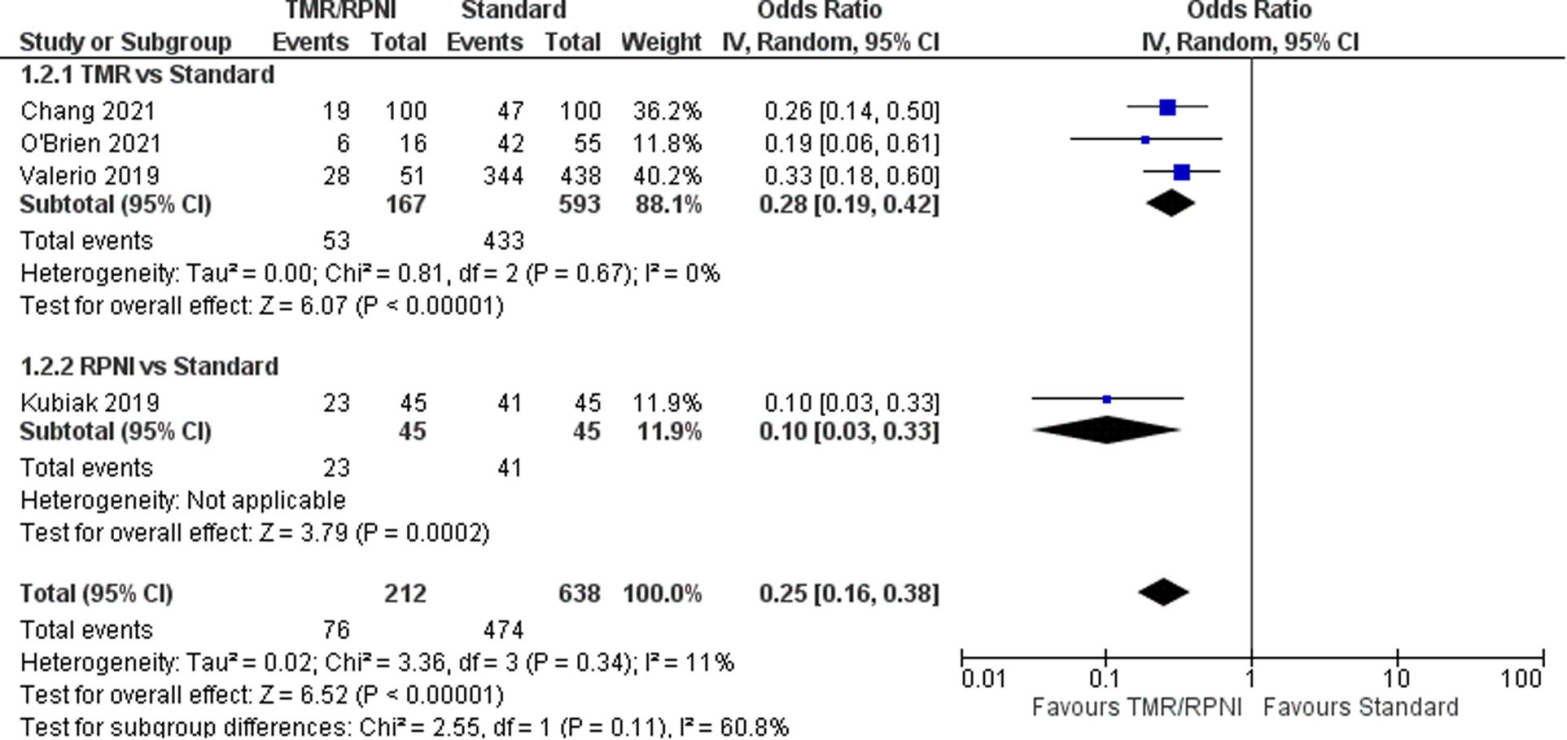
Forest Plot of Phantom Limb Pain.

For the outcome of PLP, four studies^21–24^ were included which represent 885 patients (Figure 3). The OR was calculated to be 0.25 (95% CI 0.16 to 0.38). There was moderate risk of bias in the identified study due to potential bias in confounding and measurement of outcome. The overall certainty of evidence is moderate to low. In summary, the evidence suggests that primary TMR likely results and RPNI may result in a large reduction in the incidence of PLP.

Additionally, one cohort^25^ study compared PLP in primary TMR/RPNI and secondary TMR/RPNI. They found that 6/12 patients (50%) in the primary cohort and 9/12 patients (75%) in the secondary cohort developed PLP. No statistical tests were used to assess this difference between groups. This study was not included in quantitative synthesis as the comparator group was secondary TMR/RPNI. Two case series^28,30^ identified PLP in TMR/RPNI patients only. In total, these seven studies represented 235 patients, with a pooled PLP incidence in TMR/RPNI patients of 37.9% (individual study incidence range: 19% to 75%).

### Residual Limb Pain

**Figure 4.**
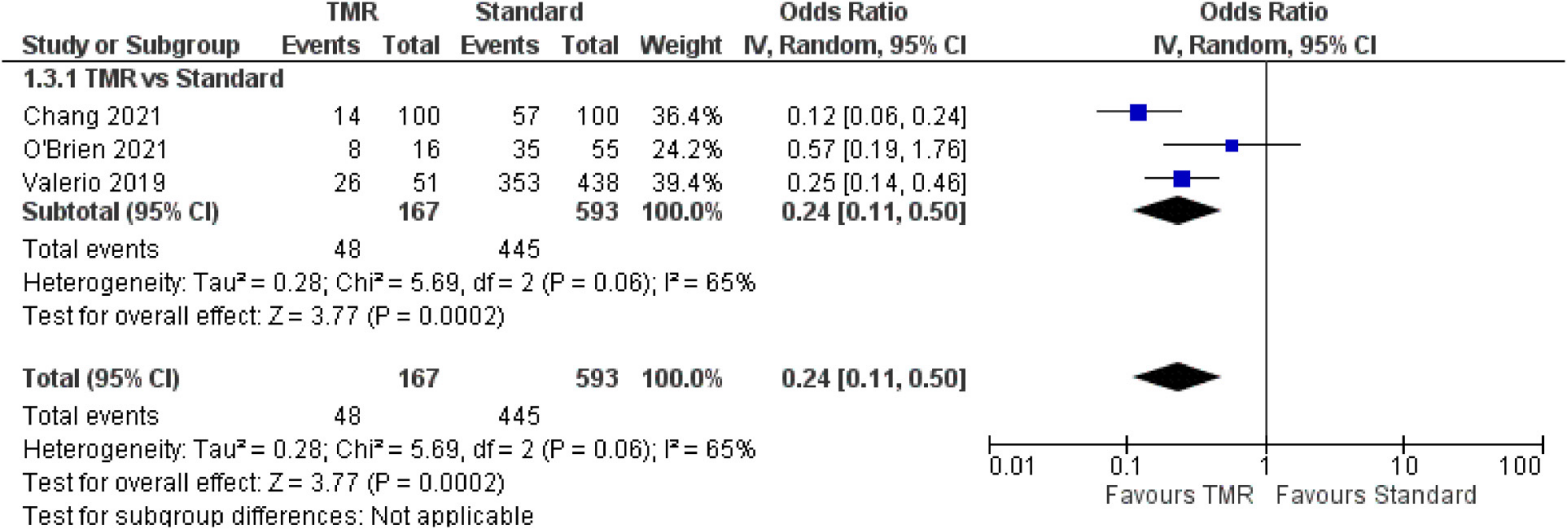
Forest Plot of Residual Limb Pain.

For the outcome of RLP, three studies^21,23,24^ were included which represent 760 patients (Figure 4). The OR was calculated to be 0.24 (95% CI 0.11 to 0.50). There was moderate risk of bias in the identified study due to potential bias in confounding and measurement of outcome. The overall certainty of evidence is low. In summary, the evidence suggests that primary TMR may result in a large reduction in the incidence of RLP.

Additionally, one case series^28^ identified RLP in TMR/RPNI patients only. In total, these four studies represented 171 patients, with a pooled RLP incidence in TMR/RPNI patients of 29.8% (individual study incidence range: 14% to 75%).

### Pain

**Figure 5.**
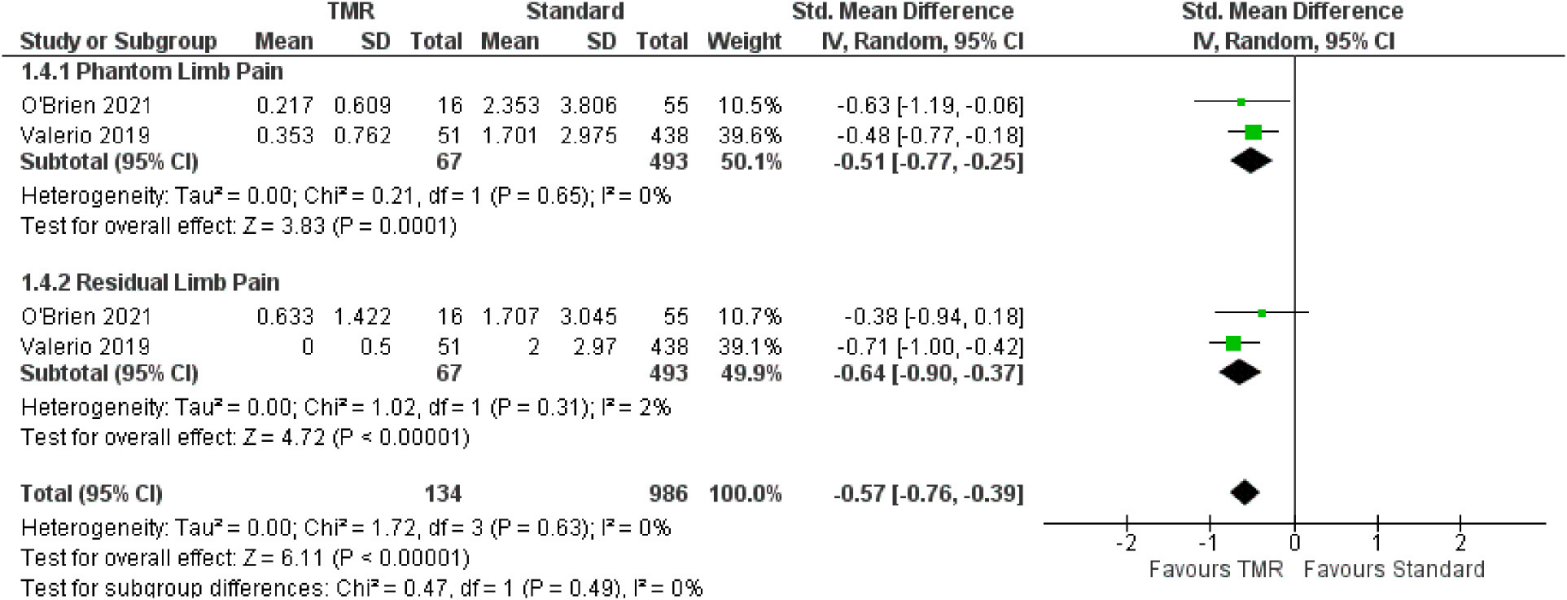
Forest Plot of Pain Scores.

For the outcome of pain, two studies were included which represent 1120 patients (Figure 5). The OR for overall pain was calculated to be −0.57 (95% CI −0.76 to −0.39). Subgroup analysis demonstrated similar ORs for PLP of −0.51 (95% CI −0.77 to −0.25) and RLP of −0.64 (95% CI −0.90 to −0.37). There was moderate risk of bias in the identified study due to potential bias in confounding and measurement of outcome. The overall certainty of evidence is low. In summary, the evidence suggests that primary TMR/RPNI may reduce PLP and RLP.

Additionally, one case series^28^ reported on pain scores. Stoehr et al reported that among four patients undergoing primary TMR at time of amputation for CRPS, the current severity of PLP was 2.0 (SD 2.4) and RLP was 5.0 (SD 4.2). This is in contrast to the worst severity in PLP of 3.8 (SD 4.3) and RLP of 6.8 (SD 4.6).

### Patient Reported Outcomes - PROMIS Scores

**Figure 6.**
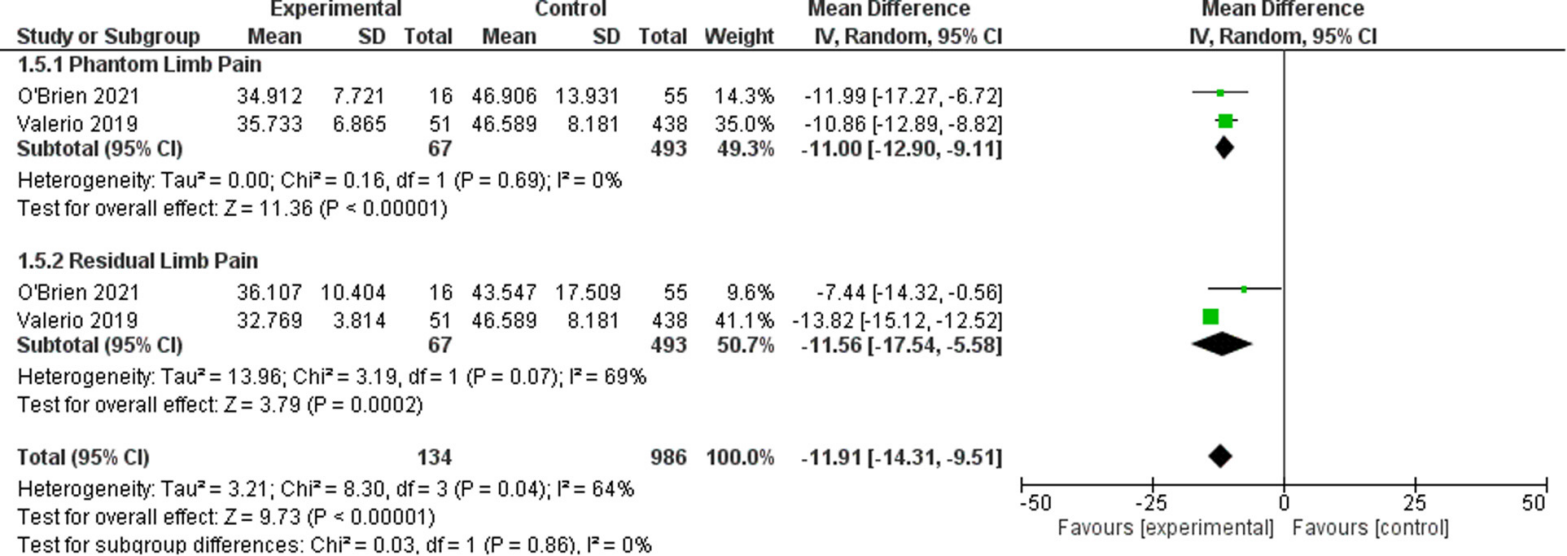
Forest Plot of PROMIS Intensity Scores.

**Figure 7.**
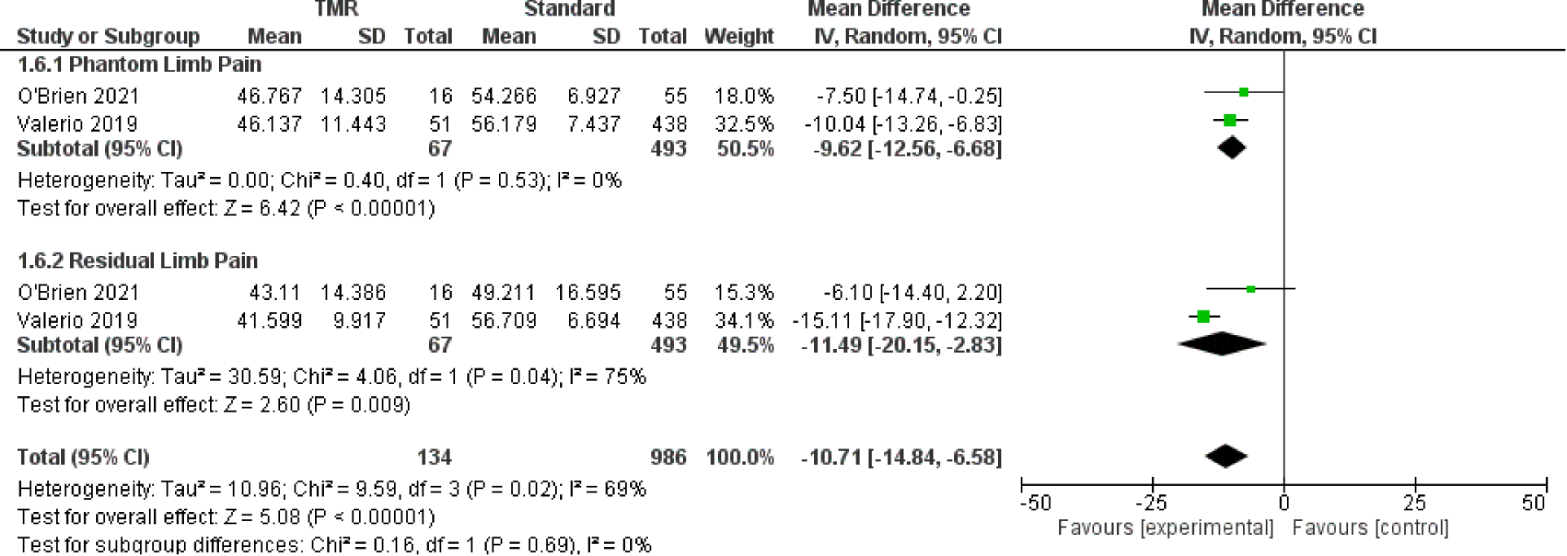
Forest Plot of PROMIS Behavior Scores.

**Figure 8.**
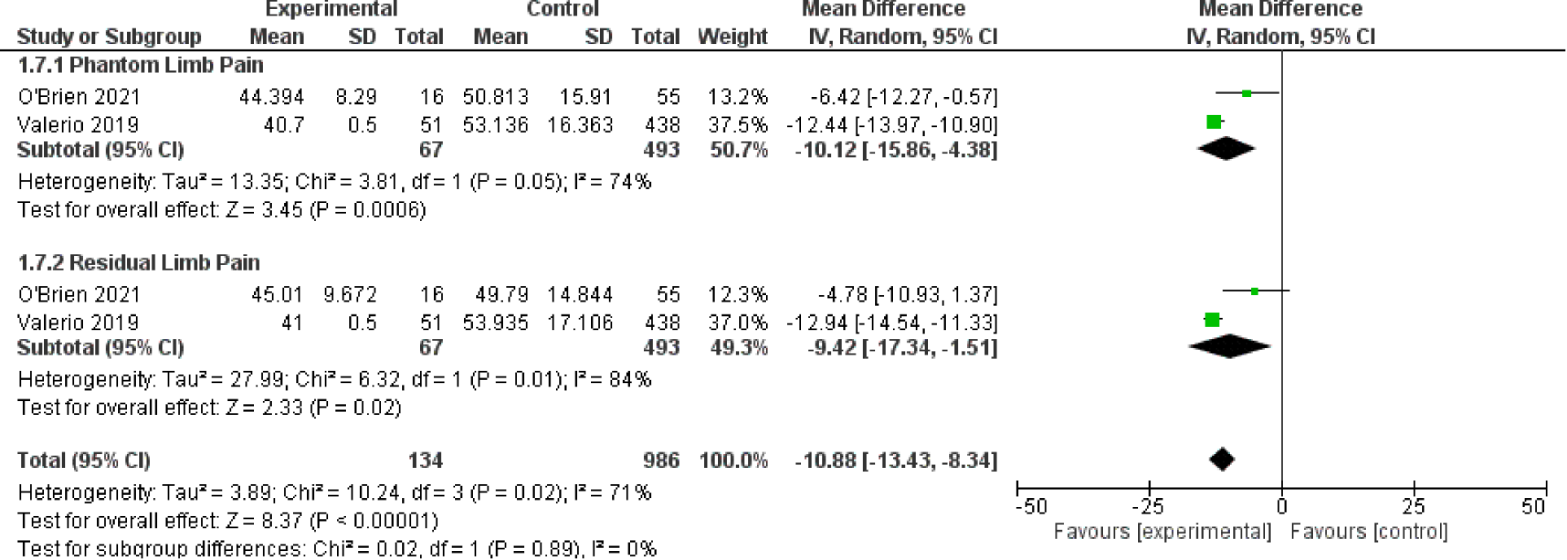
Forest Plot of PROMIS Interference Scores.

For the outcome of PROMIS scores, two studies^21,23^ were included which represent 1120 patients. The PROMIS scale has three subscales in Intensity, Behavior, and Interference, which are represented in Figures 6 to 8 respectively. The OR was calculated to be −11.91 (95% CI −14.31 to −9.51) for PROMIS Intensity, −11.91 (95% CI −14.31 to −9.51) for PROMIS Intensity, −10.71 (95% CI −14.84 to −6.58) for PROMIS Behavior. There was moderate risk of bias in the identified study due to potential bias in confounding and measurement of outcome. The overall certainty of evidence is moderate to low. In summary, the evidence suggests that primary TMR/RPNI likely reduces PROMIS Intensity and behavior scores and may reduce PROMIS Interference scores for PLP. The evidence also suggests that primary TMR/RPNI may reduce PROMIS scores in RLP.

Additionally, one cohort study^26^ and two case series^28,29^ assessed patient pain in primary TMR/RPNI using PROMIS scores. Alexander et al found significantly lower PROMIS scores in primary TMR patients compared to standard oncologic amputees in all three domains for both PLP and RLP. Data for each of the study groups was not available and could not be quantitatively synthesized.

## Discussion

To the authors’ knowledge, this is the first systematic review and meta-analysis to evaluate outcomes comparing primary TMR/RPNI and either standard treatment or secondary TMR/RPNI. Overall, the observational study evidence suggests that TMR/RPNI results in a statistically significant reduction in incidence, pain scores and PROMIS scores of PLP and RLP. Moreover, observational study evidence suggests that TMR/RPNI results in a reduction of the incidence of neuroma, but this did not achieve statistical significance (p = 0.07). No randomized data was available for this study. The GRADE evidence profile showed low to moderate certainty of evidence (Table 2).

**Table 2.** GRADE evidence of included studies.

**Summary of findings:**						
**TMR/RPNI compared to Standard for Extremity Amputation**						
**Patient or population:** Lower Extremity Amputation **Setting:** All settings **Intervention:** TMR/RPNI **Comparison:** Standard						
Outcomes	Anticipated absolute effects^*^ (95% CI)		Relative effect (95% CI)	№ of participants (studies)	Certainty of the evidence (GRADE)	Comments
	**Risk with Standard**	**Risk with TMR/RPNI**				
Incidence of Neuroma Formation	13 per 100	**1 per 100** (0 to 16)	**OR 0.07** (0.00 to 1.22)	90 (1 observational study)	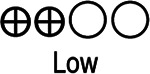	TMR/RPNI may result in a large reduction in incidence of Neuroma Formation.
Incidence of Phantom Limb Pain - TMR versus Standard	73 per 100	**43 per 100** (34 to 53)	**OR 0.28** (0.19 to 0.42)	760 (3 observational studies)	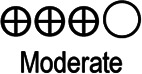	TMR/RPNI likely results in a large reduction in incidence of Phantom Limb Pain.
Incidence of Phantom Limb Pain - RPNI versus Standard	91 per 100	**51 per 100** (24 to 77)	**OR 0.10** (0.03 to 0.33)	90 (1 observational study)	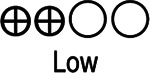	TMR/RPNI may result in a large reduction in incidence of Phantom Limb Pain.
Incidence of Residual Limb Pain - TMR versus Standard	75 per 100	**42 per 100** (25 to 60)	**OR 0.24** (0.11 to 0.50)	760 (3 observational studies)	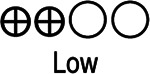	TMR/RPNI may result in a large reduction in incidence of Residual Limb Pain.
Pain Score - Phantom Limb Pain	-	SMD **0.51 lower** (0.77 lower to 0.25 lower)	-	560 (2 observational studies)	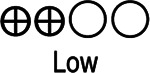	TMR/RPNI may reduce Phantom Limb Pain.
Pain Score - Residual Limb Pain	-	SMD **0.64 lower** (0.9 lower to 0.37 lower)	-	560 (2 observational studies)	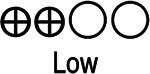	TMR/RPNI may reduce Residual Limb Pain.
PROMIS Intensity - Phantom Limb Pain	The mean PROMIS Intensity - Phantom Limb Pain was **0**	MD **11 lower** (12.9 lower to 9.11 lower)	-	560 (2 observational studies)	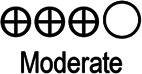	TMR/RPNI likely reduces PROMIS Intensity score for Phantom Limb Pain.
PROMIS Intensity - Residual Limb Pain	The mean PROMIS Intensity - Residual Limb Pain was **0**	MD **11.56 lower** (17.54 lower to 5.58 lower)	-	560 (2 observational studies)	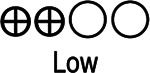	TMR/RPNI may reduce PROMIS Intensity score for Residual Limb Pain.
PROMIS Behavior - Phantom Limb Pain	The mean PROMIS Behavior - Phantom Limb Pain was **0**	MD **9.62 lower** (12.56 lower to 6.68 lower)	-	560 (2 observational studies)	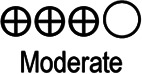	TMR/RPNI probably results in a reduction in PROMIS Behavior score for Phantom Limb Pain.
PROMIS Behavior - Residual Limb Pain	The mean PROMIS Behavior - Residual Limb Pain was **0**	MD **11.49 lower** (20.15 lower to 2.83 lower)	-	560 (2 observational studies)	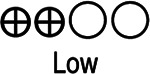	TMR/RPNI may result in a reduction in PROMIS Behavior score for Residual Limb Pain.
PROMIS Interference - Phantom Limb Pain	The mean PROMIS Interference - Phantom Limb Pain was **0**	MD **10.12 lower** (15.86 lower to 4.38 lower)	-	560 (2 observational studies)	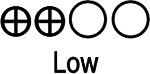	TMR/RPNI may result in a reduction in PROMIS Interference score for Phantom Limb Pain.
PROMIS Interference - Residual Limb Pain	The mean PROMIS Interference - Residual Limb Pain was **0**	MD **9.42 lower** (17.34 lower to 1.51 lower)	-	560 (2 observational studies)	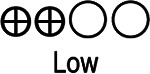	TMR/RPNI may result in a reduction in PROMIS Interference score for Residual Limb Pain.

* **The risk in the intervention group** (and its 95% confidence interval) is based on the assumed risk in the comparison group and the **relative effect** of the intervention (and its 95% CI).

**CI:** confidence interval; **MD:** mean difference; **OR:** odds ratio; **SMD:** standardised mean difference

**GRADE Working Group grades of evidence**

**High certainty:** we are very confident that the true effect lies close to that of the estimate of the effect.

**Moderate certainty:** we are moderately confident in the effect estimate: the true effect is likely to be close to the estimate of the effect, but there is a possibility that it is substantially different.

**Low certainty:** our confidence in the effect estimate is limited: the true effect may be substantially different from the estimate of the effect.

**Very low certainty:** we have very little confidence in the effect estimate: the true effect is likely to be substantially different from the estimate of effect.

**Figure 9.**
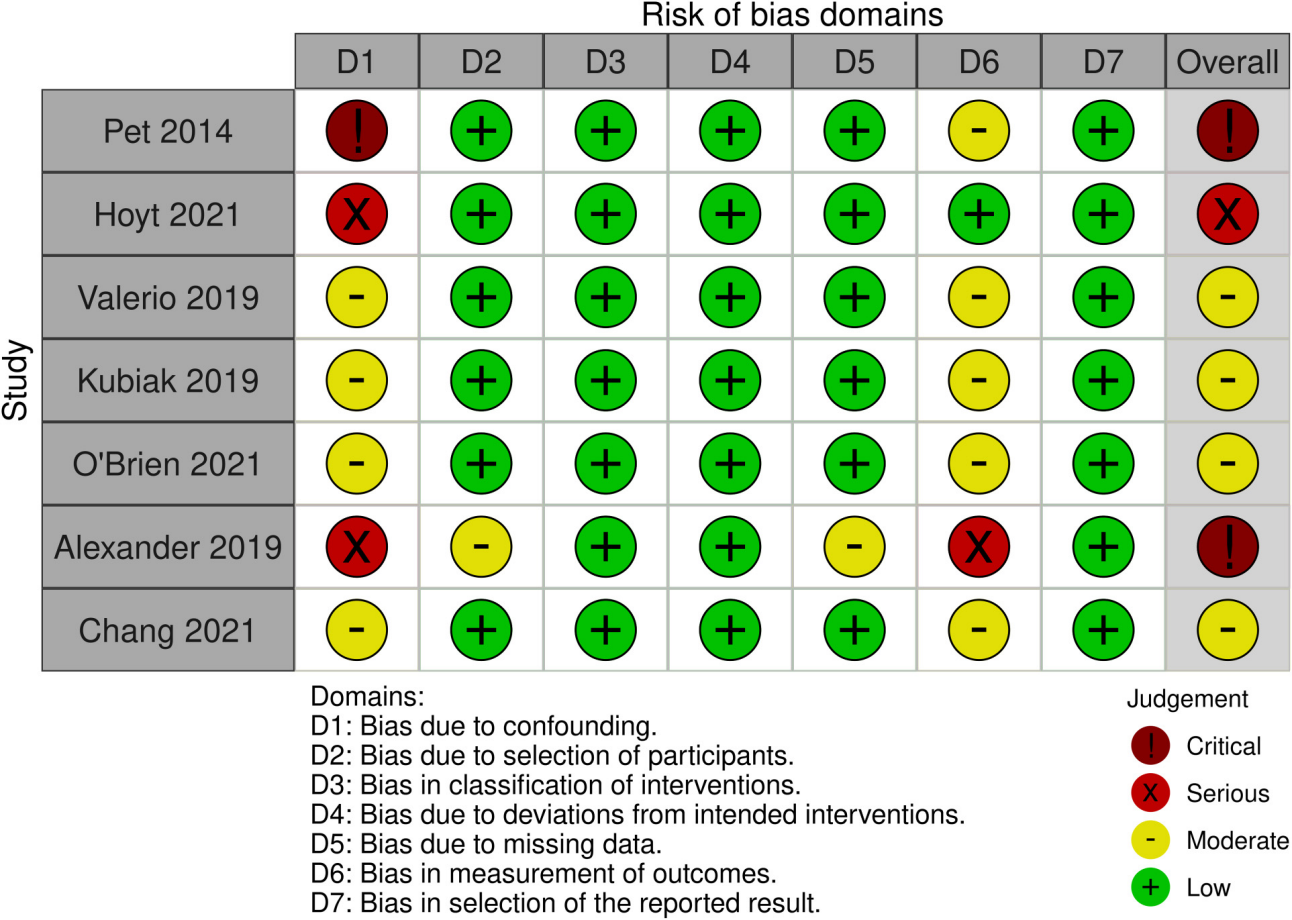
ROBINS-I Risk of Bias Assessment of Included Observational Studies.

Risk of bias assessment using the ROBINS-I tool demonstrated significant concerns for potential bias within included non-randomized studies (Figure 9). The authors noted critical risk of bias due to confounding and moderate risk of bias due to measurement of outcomes. Specifically, all primary studies did not attempt for control for baseline confounders between groups. For example, Hoyt et al provided little demographic data, and did not compare or use statistical methods to account for differences in confounders. This led to a judgment that the risk of bias in confounding was serious. There was also limited evidence to support the blinding of outcome assessors to participant intervention status, as most included studies had a retrospective design. Future research on this topic should account for key confounding factors between groups and ensure blinding of outcome assessors using a prospective design to limit the potential for bias.

This meta-analysis is in-keeping with results of existing systematic reviews comparing primary TMR/RPNI. de Lange et al^31^ performed a systematic review assessing surgical methods during amputation for neuropathic pain prevention, including finger and major limb amputations. They found that the prevention group had lower incidences than the control group in both neuropathic pain (prevention 0%-51%, control 13%-81%) and PLP (prevention 0%-56%, control 64%-91%). While our study has similar findings with reductions in neuroma and PLP in primary TMR/RPNI, only one study was available to compare incidence neuromas that did not achieve statistical significance and the reduction in incidence of PLP had moderate certainty. A systematic review abstract by He at al.^32^ investigated assessment tools for postamptuation pain and existing results of TM.They found statistically significant improvements in neuropathic pain, incidence of PLP, and PROMIS scores favoring primary TMR/RPNI compared to standard amputations, but not between secondary TMR/RPNI and standard amputations. The results of this data supports our findings, as we report similar outcomes, and results in favor of primary TMR/RPNI.

Several limitations were identified in this study. Gray literature was not incorporated in the search strategy, which reduces the available evidence and may introduce publication bias. Due to the inclusion of less than 10 studies for quantitative synthesis, a funnel plot could not be constructed and assessment of publication bias was not performed. Furthermore, there was no randomized trial data available, with most included studies for quantitative analysis utilizing a retrospective design, which limited the certainty of evidence. Future research should utilize prospective designs to reduce the risk of bias. For example, an RCT was performed by Dumanian et al^33^ evaluating secondary TMR and standard treatment in chronic postamputation pain. A similar study design can be adapted to evaluate primary TMR/RPNI outcomes. For the purposes of performing a meta-analysis, the present study pooled outcomes for TMR and RPNI. This pooled analysis assumes that both TMR and RPNI improve on outcomes as compared to standard treatment. As such, the findings may not represent the efficacy of each approach if the two techniques do not produce the same outcomes. Finally, there was limited evidence available with low to moderate certainty in findings, and therefore should be interpreted with caution.

## Conclusion

Overall, the observational data suggests that primary TMR/RPNI likely reduces incidence, pain scores and PROMIS scores of PLP and RLP. The data also suggests that primary TMR/RPNI may reduce incidence of neuromas, but this did not achieve statistical significance. Surgeons should weigh the uncertainty of evidence with potential harms and benefits when considering the use of primary TMR/RPNI in the surgical management of major limb amputations. Going forward, randomized trials are warranted to evaluate this research question, particularly to improve the certainty of evidence.

## Supplemental Material

sj-docx-1-psg-10.1177_22925503221107462 - Supplemental material for Targeted Muscle Reinnervation and Regenerative Peripheral Nerve Interfaces Versus Standard Management in the Treatment of Limb Amputation: A Systematic Review and Meta-Analysis Supplemental material, sj-docx-1-psg-10.1177_22925503221107462 for Targeted Muscle Reinnervation and Regenerative Peripheral Nerve Interfaces Versus Standard Management in the Treatment of Limb Amputation: A Systematic Review and Meta-Analysis by Morgan Yuan, Matteo Gallo, Lucas Gallo, Minh HQ Huynh, Mark McRae, Matthew C. McRae, Achilleas Thoma, Christopher J. Coroneos and Sophocles H. Voineskos in Plastic Surgery
