# Supplementary material for: Targeted Muscle Reinnervation and Regenerative Peripheral Nerve Interfaces Versus Standard Management in the Treatment of Limb Amputation: A Systematic Review and Meta-Analysis
Source: Plast Surg (Oakv). 2022 Jun 16;32(2):253–64. doi: 10.1177/22925503221107462 (PMC11046287; doi:10.1177/22925503221107462)
Supplement: sj-docx-1-psg-10.1177_22925503221107462 - Supplemental material for Targeted Muscle Reinnervation and Regenerative Peripheral Nerve Interfaces Versus Standard Management in the Treatment of Limb Amputation: A Systematic Review and Meta-Analysis [file sj-docx-1-psg-10.1177_22925503221107462.docx]

**Supplementary Material 1. Search Strategy**

**Medline**

exp Amputation/

amput*.ti,ab,kf,kw.

1 or 2

reinnervat*.ti,ab,kf,kw.

Regenerative peripheral nerve interface.mp.

Nerve regeneration/

((nerve* or neural or nervous) adj2 regenerat*).ti,ab,kf,kw.

4 or 5 or 6 or 7

3 and 8

**Embase**

exp Amputation/

amput*.ti,ab,kw.

1 or 2

reinnervat*.ti,ab,kw.

Regenerative peripheral nerve interface.mp.

Nerve regeneration/

((nerve* or neural or nervous) adj2 regenerat*).ti,ab,kw.

4 or 5 or 6 or 7

3 and 8

**Central**

#1 MeSH descriptor: [Amputation] explode all trees

#2 (“amputate”):ti,ab,kw

#3 (“amputation”):ti,ab,kw

#4 (“amputee”):ti,ab,kw

#5 (#1 or #2 or #3 or #4)

#6 (“TMR”):ti,ab,kw

#7 (“targeted muscle reinnervation”):ti,ab,kw

#8 (#6 or #7)

#9 (#5 and #8)
